# SCI – U: Validation of an updated Self-Care Inventory for contemporary diabetes management in adolescents with type 1 diabetes

**DOI:** 10.1016/j.diabres.2025.112218

**Published:** 2025-04-30

**Authors:** Sarah S. Jaser, Tamim A. Hamdan, Marisa E. Hilliard, Joyce P. Yi-Frazier, Randi Streisand, Faisal S. Malik, Daniel J. DeSalvo, Maeve B. O’Donnell, Annette M. La Greca

**Affiliations:** a Vanderbilt University Medical Center, Nashville, TN, United States; b Baylor College of Medicine and Texas Children’s Hospital, Houston, TX, United States; c University of Washington School of Medicine and Seattle Children’s Research Institute, Seattle, WA, United States; d George Washington School of Medicine and Health Sciences and Children’s National Hospital, Washington, DC, United States; e University of Washington Tacoma, Tacoma, WA, United States; f University of Miami, Coral Gables, FL, United States

**Keywords:** Type 1 Diabetes, Self-Care Behavior, Measurement, Technology and Diabetes, Continuous Glucose Monitoring (CGM), Questionnaire Validation

## Abstract

**Aims:**

The Self-Care Inventory is a widely used measure to assess diabetes self-management behaviors. We sought to adapt and streamline the measure to reflect advances in diabetes management, including increased use of continuous glucose monitor and automated insulin delivery systems.

**Methods:**

Through an expert-driven, iterative process, the updated Self-Care Inventory (SCI-U) was created by modifying items to reflect modern diabetes management and reducing the measure to 8 items. The measure was administered to 369 adolescents with type 1 diabetes in 4 regions of the United States, at baseline of two separate intervention trials (mean age = 15.5 ± 1.5, 56 % female, 59 % non-Hispanic White) with measures of diabetes self-management and health-related quality of life. Data on adolescents’ device usage and glycemic outcomes were also collected.

**Results:**

The SCI-U demonstrated good reliability (*α* = 0.68 for the adolescent self-report version and 0.75 for the caregiver proxy version) and construct validity; it was significantly associated with other measures of diabetes self-management (Diabetes Self-Management Questionnaire, percentage of time continuous glucose monitor was active), and criterion validity with diabetes-related quality of life, HbA1c, and time in range.

**Conclusions:**

The updated, shortened SCI-U is a reliable, valid measure for assessment of diabetes self-management behaviors in adolescents with type 1 diabetes.

## 1. Introduction

Problems with diabetes management and difficulty meeting glycemic targets are common among adolescents with type 1 diabetes and are strongly linked with poor long-term health outcomes [1]. Efforts to promote self-management behaviors are critical and require sound assessment tools. Yet, measuring diabetes self-management behaviors is particularly challenging because the self-management requirements (e. g., blood glucose monitoring, nutrition and activity recommendations, insulin administration) vary across individuals, even among people of the same age with the same type of diabetes [1]. While diabetes devices, such as insulin pumps or continuous glucose monitor (CGM) systems offer objective data on specific self-management behaviors (e.g., device wear time, frequency of insulin bolus administration), not all adolescents use this technology, and comprehensive assessment of self-management behaviors across multiple domains often relies on self-report. No “gold standard” measure exists for type 1 diabetes self-management behaviors, but prior studies support the use of self- and parent-reports of diabetes self-management behaviors in adolescents [2,3]. A brief, reliable, and valid measure of these behaviors is needed for clinical purposes and research (i.e., to demonstrate efficacy of interventions).

The Self-Care Inventory (SCI) is a flexible tool for assessing diabetes self-management behaviors. The SCI was originally developed and tested in adolescents with type 1 diabetes and their caregivers [4,5] and later modified for use in adults with diabetes [6]. While other measures of diabetes self-management behaviors exist [7–9], these measures have limitations, including not reflecting current diabetes management and length/response burden. Structured interviews [10,11] also provide an opportunity for more detailed assessment, but they are lengthy and often not practical for use in clinical research and practice settings. Thus, a brief self-report measure is needed.

The original SCI consists of 14 items that ask about engaging in specific diabetes self-management behaviors (i.e., “Please rate each of the items according to how well you/your child followed your prescribed regimen for diabetes care in the past month”). The SCI demonstrated strong reliability and validity, including associations with hemoglobin A1c (HbA1c) and other key diabetes outcomes, and quickly became one of the most widely used measures of adherence and diabetes management in children and adolescents. In 2004, the manual for the SCI recommended administering the full measure for clinical purposes but using only 7 of the 14 items for an overall score [4].

In 2009, the psychometric properties of the adolescent and parent SCI forms were evaluated in a sample of adolescents ages 11–18 using different insulin regimens (i.e., insulin pump, multiple daily injections with insulin aspart/lispro and glargine, NPH/regular, or pre-mixed insulin) [12]. For this study, all 14 items were considered in the total score (omitting those which participants indicated were not applicable). The adolescent and parent forms demonstrated sufficient internal consistency (*α* = 0.80, 0.72, respectively), moderate parent–child agreement (ICC = 0.47), and strong test–retest reliability (*r* = 0.91 for adolescents, *r* = 0.86 for parents, both *p* < 0.001) [12]. While the wording of items was appropriate for a sample of adolescents with type 1 diabetes in 2009 (i.e., could be applied to those using insulin pumps, only 14 % of the sample), the items focused on blood glucose monitoring were more applicable for adolescents using glucose meters (e.g., glucose testing, glucose recording) than current glucose monitoring devices used to support diabetes management.

Although the SCI continues to be a useful measure, its relevance and face validity are threatened as diabetes technology advances. Current standards of care include offering CGM and insulin pumps – including automated insulin delivery systems (AID) – starting at the time of diagnosis [1]. As such, the uptake of CGM and insulin pumps has risen dramatically among adolescents in the past 5 years [13]. In a study of 21 U.S. pediatric diabetes centers participating in the Type 1 Diabetes Exchange Quality Improvement Collaborative [14], median CGM use across sites was 81 % and insulin pump use was 52 % (as of 2022), and current rates are likely higher. Thus, some items in the original SCI may no longer be applicable, and an update of the SCI is needed to assess modern diabetes self-management behaviors more accurately, without being limited to specific technologies. There is also a need to enhance the assessment’s efficiency by omitting items that are no longer relevant.

In the current study, we evaluated the psychometric properties of the newly updated SCI (SCI-U) in a large sample of adolescents with type 1 diabetes and their caregivers. We hypothesized that the SCI-U would demonstrate acceptable reliability (defined as internal consistency, α), construct validity (defined as correlations with other measures of self-management), and criterion validity (defined as correlations with diabetes-related quality of life and glycemic outcomes).

## 2. Material and methods

### 2.1. SCI-U revision

The SCI underwent an iterative revision process by a team of experts, including specialists in pediatric endocrinology, diabetes self-management behaviors, and questionnaire development. This team, including an author of the original SCI (AML), reviewed the original 14 items and made revisions to align with contemporary type 1 diabetes management. These revisions included two additional questions specific to insulin adjustment and glucose data review. Table 1 documents the modifications by comparing items from the original SCI with items from the new SCI-U. This process resulted in a 9-item SCI-U, and a caregiver proxy report was created using the same items (see Supplemental Table). The SCI-U retained the original instructions and response options as the previous versions: “Please rate each of the items according to how well you/your child followed your prescribed regimen for diabetes care in the past month, on a scale of 1 (*Never do it*) to 5 (*Always do this as recommended without fail*).” For items that are not relevant, individuals can choose “N/A.” Mean scores are calculated; scores range from 1 to 5, with higher scores indicating higher self-management behaviors.

The current study includes baseline data from participants enrolled in two separate randomized controlled trials aimed at reducing diabetes distress in adolescents, PRISM and THR1VE!, from 2019 to 2022 [15,16]. Eligibility criteria included a diagnosis of type 1 diabetes for at least one year, elevated diabetes distress as reported on the Problems Areas in Diabetes-Teen measure [17], and fluency in English. Baseline data were collected prior to participants receiving interventions. All youth participants completed the SCI-U (n = 369), and caregivers of youth ≤ 17 years (n = 326) completed the SCI-U proxy report.

### 2.2. Construct validity measures

#### 2.2.1. Diabetes Self-Management Questionnaire

The Diabetes Self-Management Questionnaire (DSMQ) is a validated 9-item questionnaire assessing engagement in diabetes self-management tasks [9]. Adolescents in the PRISM study completed a self-report measure (n = 171), and caregivers (n = 131) completed a proxy measure. Higher scores indicate higher engagement. In the current sample, Cronbach’s alpha was 0.61 for the adolescent measure and 0.64 for the caregiver proxy measure.

#### 2.2.2. Active CGM use

For participants who were using a CGM at the time of study enrollment (n = 285), we obtained data from device uploads. Glucose readings from CGM devices are measured every ~ 1–5 min, and device data reports were shared with the diabetes clinics/electronic health record or the research teams. Percentage of time CGM was active is an objective measure of diabetes self-management, as it captures the proportion of time the CGM was active for the number of days a CGM device is worn (recommend ≥ 70 % of data from 14 days) [18]. Higher scores on the SCI-U were expected to correlate with higher percentage of time CGM was active.

### 2.3. Criterion validity measures

#### 2.3.1. Health-Related quality of life

The Type 1 Diabetes and Life (T1DAL) measure is a validated self-report measure of diabetes-specific health-related quality of life (HRQoL) designed to examine a range of aspects of living with type 1 diabetes across the lifespan [19]. Participants completed the appropriate T1DAL version based on their age at consent: adolescents aged 17 and below completed the 23-item T1DAL-Teen, and those aged 18 years completed the 27-item T1DAL-Young Adult [20]. Caregivers of participants up to age 17 completed the 30-item T1DAL-Parent version to assess their own experience [21]. All versions are scored on a 0–100 scale, with higher scores indicating better HRQoL. Subscales for each version differ, so only total scores are reported here. A positive correlation between the T1DAL and SCI-U was hypothesized, as was demonstrated with the SCI-R in the T1DAL validation studies. In this sample, internal consistency was good, α = 0.83 for adolescents, 0.78 for young adults, and 0.87 for caregivers, respectively.

#### 2.3.2. Hemoglobin A1c

HbA1c reflects an individual’s average blood glucose level over 2–3 months prior to its measurement, and it is routinely assessed during regular diabetes clinic visits [1]. Participants’ HbA1c results were obtained from medical charts to ensure accuracy. For participants who did not have point-of-care HbA1c levels documented from clinical care (e.g., due to telehealth visits during the pandemic), dried blood spot HbA1c tests were used that were completed at home [22]. The American Diabetes Association recommends a HbA1c target of less than 7 % (53 mmol/mol) for most people with type 1 diabetes [1]. HbA1c was hypothesized to negatively correlate with the SCI-U, as was the case with the original SCI [12].

#### 2.3.3. Time in range

Time in range (TIR) is the percentage of time an individual’s glucose levels were within their target glucose range (typically 70–180 mg/dL) over 14 days. The American Diabetes Association recommends a target TIR of ≥ 70 % (19). Participant TIR percentages were directly obtained from CGM reports and were hypothesized to positively correlate with the SCI-U.

### 2.4. Statistical analyses

Descriptive analyses were conducted separately for the adolescent and caregiver proxy measures, including a measure of skewness. Intraclass correlations were obtained as an estimate of internal consistency. Paired t-tests and bivariate correlations were conducted to determine correspondence between adolescent and caregiver proxy reports. Pearson correlations were conducted to evaluate construct validity (associations between the SCI-U and the DSMQ and Percent Active) and criterion validity (associations between the SCI-U and measures of quality of life and glycemic outcomes of HbA1c and TIR).

## 3. Results

### 3.1. Participants

The combined sample included 369 adolescents, ages 13–19, and 326 caregivers recruited across pediatric diabetes clinics in four regions of the United States: Pacific Northwest (n = 85), Gulf Coast (n = 86), Southeast (n = 109), and Mid-Atlantic (n = 89). Table 2 details the sample’s demographic and clinical characteristics. Of note, only 16 % of adolescents were meeting the target for HbA1c (<7%, or 53 mmol/mol), and 16 % were meeting the target for TIR (≥70 %).

### 3.2. SCI-U Descriptive Statistics

Based on the initial review of item-level data, indicating that Item #2 (*Record or upload blood glucose data*) was most likely to be marked “N/A” and would not reduce reliability if deleted (see Supplemental Table), we conducted analyses on the 8-item version of the scale.

For the adolescent version of the SCI-U, the mean was 3.66 ± 0.57 and median was 3.75 (IQR: 3.33, 4.13). Scores ranged from 1.63 to 4.88, and skewness was −0.62, SE = 0.13, indicating the data were more frequently at the high end of the score range. For the caregiver proxy version of the SCI-U, the mean was 3.60 ± 0.59 and median was 3.63 (IQR: 3.25, 4.00). Scores ranged from 1.75 to 4.88, and skewness was −0.38, SE = 0.14, indicating the data were more frequently at the high end of the score range.

### 3.3. Reliability

The internal consistency estimate was *α* = 0.68 for adolescent self-report and *α* = 0.75 for caregiver proxy-report.

### 3.4. Adolescent-Caregiver correspondence

The correlation between adolescent self-report and caregiver-report was *r* = 0.40 (p < 0.001), indicating moderate correspondence. The paired *t*-test between adolescent and caregiver reports was also significant (t = −2.11, p = 0.04); adolescents reported a higher level of self-management behaviors than did caregivers within dyads.

### 3.5. Validity

Table 3 shows correlations between the SCI-U adolescent and caregiver measures and the measures of construct and criterion validity. A positive correlation was found between adolescents’ self-reported SCI-U and both the adolescent and caregiver version of the DSMQ (*r* = 0.55, 0.18, respectively, both *p* < 0.05). Similarly, a positive correlation was found with the caregiver SCI-U proxy report and the adolescent and caregiver versions of the DSMQ (*r* = 0.40, 0.43, respectively, both *p* < 0.001). In addition, adolescents’ and caregivers’ scores on the SCI-U were significantly associated with greater percentage of active time on CGM (*r* = 0.16, 0.15, respectively, both p < 0.05).

Adolescent SCI-U score was significantly associated with higher self-reported diabetes-related quality of life (*r* = 0.31, p < 0.001), and caregiver proxy-reported SCI-U score was significantly associated with higher caregiver self-reported diabetes-related quality of life (*r* = 0.37, p < 0.001). SCI-U scores were also significantly associated with glycemic outcomes. Specifically, higher SCI-U was associated with lower HbA1c levels (adolescent: *r* = −0.17; caregiver: 0.16; both *p* < 0.01). Among the n = 238 with CGM data available for analysis, higher SCI-U was associated with higher TIR (adolescent: *r* = 0.27; caregiver: 0.39; both *p* < 0.001).

## 4. Discussion

The updated, shortened SCI-U is a psychometrically sound measure of self-management behaviors for adolescents with type 1 diabetes. The SCI-U demonstrated good reliability, as well as strong construct validity with another measure of diabetes self-management (DSMQ) and a more objective indicator of diabetes management (CGM percent active time). Combining data from two separate randomized controlled trials conducted across four distinct regions of the United States generated a sample of adolescents and caregivers from a range of racial, ethnic, socioeconomic, and geographic backgrounds to validate this updated measure. Of note, the current study included a larger and more diverse sample than used in prior validation studies of the SCI (e.g., Lewin et al. 2009 sample was n = 164, 72 % non-Hispanic White) [22]. The updated measure’s solid psychometric properties within this sample are encouraging; we found remarkable similarities in the psychometric properties of the measure across the study sample.

The correspondence between adolescent and caregiver report of adolescent self-management behavior was moderate (*r* = 0.40) but significant and in line with the correspondence between child and caregiver report on the earlier version of the SCI [12]. Adolescents’ and caregivers’ scores were also significantly associated with reports of their own health-related quality of life. This may suggest that youth and caregiver views of youth self-management behaviors relate to how diabetes impacts their own lives, or it may be an artifact of single-informant bias. One of the strengths of the current study is that we included multiple informants (caregivers and adolescents) of adolescents’ self-management behavior. However, based on the strength of association with glycemic outcomes and HRQOL, if time is limited, adolescents’ self-report may be prioritized.

It is important to note that the current sample included adolescents with elevated diabetes distress, as the data were collected at baseline for intervention trials targeting diabetes distress. Thus, the participants completing the surveys may not reflect the experiences of all adolescents with type 1 diabetes, and validation is needed in other population-based samples. However, diabetes distress is quite common among adolescents, with over one-third of adolescents with type 1 diabetes endorsing elevated diabetes distress [23]. Thus, validation of the SCI-U in a sample with elevated diabetes distress adds to the literature by demonstrating the psychometric validity of this measure in the context of common diabetes distress.

Our sample also reflects more contemporary rates of diabetes device use (from participants enrolled from 2019 to 2022); in this combined sample, 77 % of adolescents were using CGM and 63 % were using insulin pumps. Population use rates may be even higher now, as AID systems are becoming more accessible and insurance coverage for CGM devices has become more widespread [24]. Validation of this SCI-U measure among a sample with high diabetes technology rates matches the current clinical landscape and enhances the measure’s relevance and applicability to current research and diabetes care. Future studies could evaluate the associations between self-management behaviors assessed with the SCI-U and other measures of technology use, such as the Benefit and Burdens of CGM [25] or INSPIRE measures for AID systems [26].

### 4.1. Clinical Implications

Assessing and improving diabetes self-management behaviors among adolescents are critical issues for health care providers, and initial analyses of the SCI-U support the instrument’s reliability and validity. Reducing the SCI to 8 items also added efficiency, which may lessen the burden on patients in clinical settings. The SCI-U items reflect contemporary diabetes management, which allows this measure to be used in clinical care settings to identify adolescents who may need more support for diabetes management.

### 4.2. Limitations

As with all studies that use data from clinical trials, participants who enroll in intervention studies may not reflect the larger population, including youth and families who may not have time, interest, or opportunity to participate in such research, introducing potential selection bias. Future work should also aim to assess the SCI-U’s performance in a sample of participants with diverse levels of diabetes distress. Moreover, English language fluency was a requirement for the adolescents in these trials, which excluded youth who may experience barriers to healthcare due to language. However, youth with Spanish-speaking parents were included in one of the studies, which reduced this limitation. We were not able to evaluate test–retest reliability or sensitivity to change of the updated measure in the current study, and this will be important work to establish the utility of the measure. Finally, though the items on this measure are currently up to date with current diabetes management, we acknowledge that treatment approaches and care guidelines are ever evolving, and terminology may also change over time; thus, we expect future updates may be needed.

In summary, the SCI-U is a convenient, brief, and modern tool to use for research and clinical assessment of diabetes self-management in adolescents with type 1 diabetes.

## Supplementary Material

1

## Figures and Tables

**Table 1:** Self-Care Inventory (SCI) Versions and Changes.

Original SCI		SCI-U	Reason for Change
1.	Glucose Testing*	Check glucose with monitor (including continuous glucose^+^ monitoring (CGM)^^^	Broaden methods of checking glucose values to reflect contemporary T1D management
1.	Glucose Recording*	Review glucose data^+^	Focus on active management (recording is mostly passive with current technology)
2.	Ketone Testing		Omitted due to typical infrequency of when ketone testing is needed
3.	Administering correct insulin dose	Take the correct dose of insulin	Updated to reflect contemporary management, including automated insulin delivery (AID) systems
4.	Administering insulin at right time*	Take insulin at the right time	Updated to reflect contemporary management, including AID systems
5.	Adjusting insulin intake based on blood glucose values*	Adjust insulin dosage based on glucose values, food, and exercise	Updated to reflect nuances of contemporary management
6.	Eating the proper foods; sticking to a meal plan*	Eat the correct food portions (consistent with insulin doses)	Reflects contemporary T1D management related to nutrition
7.	Eating meals on time*	Eat meals/snacks within 15 min of a bolus (i.e., taking fast-acting insulin)	Reflects contemporary T1D management related to nutrition
8.	Eating regular snacks		Combined with above
9.	Carrying quick-acting sugar to treat reactions		Omitted from original score because most people report doing this very often
10.	Coming in for appointments		Omitted from original score because most people report doing this very often
11.	Wearing a medic alert ID		Omitted from original score because most people report doing this very often
12.	Exercising regularly*	Exercise	Simplified wording to eliminate confusion around regular or strenuous
13.	Exercising strenuously		Combined with above

Note.

* Included in the scoring of the original SCI measure.

+ Changed from “blood glucose” in the version administered in the studies to “glucose” for greater accuracy.

^ Omitted “or hybrid closed loop system” from the version administered in the studies to streamline the item, since hybrid closed loop systems include CGMs.

Original SCI-U items listed in Supplemental Table.

**Table 2:** Participant Demographic and Clinical Characteristics.

**Demographic Characteristics**	**Adolescent Age, Years**, M ± SD	15.5 ± 1.5
	**Adolescent Gender**, N (%)	
	Male	148 (40 %)
	Female	201 (56 %)
	Other	13 (4 %)
	**Adolescent Race**, N (%)	
	White	256 (70 %)
	African American/Black	73 (20 %)
	Asian	15 (4 %)
	American Indian or Alaska Native	6 (2 %)
	Biracial	16 (4 %)
	Other	2 (1 %)
	**Adolescent Ethnicity**, N (%)	
	Non-Hispanic or Latinx	327 (89 %)
	Hispanic or Latinx	42 (11 %)
**Diabetes Characteristics**	**T1D Duration, Years**, M ± SD	6.5 ± 3.9
	**HbA1c, %**, M ± SD	8.9 ± 2.1
	**Meeting Target HbA1c (<7%)**, N (%)	58 (16 %)
	**Current CGM User**, N (%)	285 (77 %)
	**CGM Percent Active**, M ± SD	84 ± 20
	**Time in Range (TIR)**, %, M ± SD	46 ± 22
	**Meeting Target TIR (≥70 %)**, N (%)	39 (16 %)
	**Insulin Pump User**, N (%)	231 (63 %)
**Psychosocial Characteristics**	**Diabetes-Related Quality of Life (T1DAL)**, M ± SD	
	T1DAL Caregiver	57.9 ± 13.3
	T1DAL Adolescent/YA	49.9 ± 13.3
	**Diabetes Self-Management Questionnaire (DSMQ)**, M ± SD	
	DSMQ Caregiver	32.8 ± 6.6
	DSMQ Adolescent	20.0 ± 6.3

**Table 3:** Validity Estimates for SCI-UAdolescent and Caregiver Measures.

Scale	T1DAL Adol/YA	T1DAL Caregiver	DSMQ Adolescent	DSMQ Caregiver	Percent Active	HbA1c	Time in Range
SCI-U Adolescent	0.31***	0.11*	0.55***	0.18*	0.16*	−0.17***	0.27***
SCI-U Caregiver	0.07	0.37***	0.40***	0.43***	0.15*	−0.16**	0.39***

*Note*. DMSQ only available for the PRISM study adolescents (n = 171) and caregivers (n = 131). Percent Active (n = 212) and Time in Range (n = 238) only available for participants with CGM data. Correlations with the 9-item version administered were essentially the same, but the SCI-U adolescent measure was not significantly associated with T1DAL caregiver in the 9-item version.

* p < 0.05.

** p < 0.01.

*** p < 0.001.

## Data Availability

Data will be made available on request.

## Appendix A. Supplementary material

Supplementary data to this article can be found online at https://doi.org/10.1016/j.diabres.2025.112218.
