# Supplementary material for: SCI – U: Validation of an updated Self-Care Inventory for contemporary diabetes management in adolescents with type 1 diabetes
Source: Diabetes Res Clin Pract. Author manuscript; Available in PMC 2026 Jun 2. (PMC13229383; doi:10.1016/j.diabres.2025.112218)
Supplement: 1 [file NIHMS2175186-supplement-1.docx]

**Supplemental Table. Item-Level Data for the SCI-U Adolescent and Caregiver Measures**

| **Item** | **N** | **Mean** | **Standard Deviation** | **Item-Total Correlation** | **Cronbach’s Alpha if Item Deleted** |
| --- | --- | --- | --- | --- | --- |
| Adolescent Self-Report Measure | | | | | |
| 1. Check blood glucose with monitor (including continuous glucose monitoring (CGM) or hybrid closed loop system. | 367 | 4.18 | 0.94 | 0.26 | 0.69 |
| 1. Record or upload blood glucose data. | 351 | 2.99 | 1.38 | 0.34 | 0.68 |
| 1. Review blood glucose data. | 364 | 3.04 | 1.21 | 0.38 | 0.67 |
| 1. Take the correct does of insulin. | 367 | 4.14 | 0.75 | 0.46 | 0.67 |
| 1. Take insulin at the right time. | 368 | 3.60 | 0.89 | 0.47 | 0.66 |
| 1. Eat the correct food portions (consistent with insulin doses). | 363 | 3.43 | 0.96 | 0.49 | 0.66 |
| 1. Eat meals/snacks within 15 minutes of a bolus (i.e., taking fast-acting insulin). | 365 | 3.26 | 1.18 | 0.29 | 0.69 |
| 1. Exercise. | 366 | 3.68 | 1.16 | 0.31 | 0.69 |
| 1. Adjust insulin dosage based on glucose values, food, and exercise. | 366 | 3.98 | 1.06 | 0.48 | 0.65 |
| Caregiver Proxy Report Measure | | | | | |
| 1. My child checks blood glucose with monitor (including continuous glucose monitoring (CGM) or hybrid closed loop system). | 324 | 4.26 | 0.93 | 0.41 | 0.75 |
| 1. My child records blood glucose data. | 299 | 2.34 | 1.49 | 0.43 | 0.75 |
| 1. My child reviews blood glucose data. | 323 | 2.68 | 1.29 | 0.54 | 0.72 |
| 1. My child takes the correct dose of insulin. | 325 | 4.01 | 0.66 | 0.48 | 0.74 |
| 1. My child takes insulin at the right time. | 326 | 3.62 | 0.81 | 0.59 | 0.73 |
| 1. My child eats the correct food portions (consistent with insulin doses). | 324 | 3.47 | 0.84 | 0.50 | 0.74 |
| 1. My child eats meals/snacks within 15 minutes of a bolus (i.e., taking fast-acting insulin). | 322 | 3.51 | 1.02 | 0.42 | 0.74 |
| 1. My child exercises. | 326 | 3.50 | 1.15 | 0.25 | 0.77 |
| 1. My child adjusts insulin dosage based on glucose values, food, and exercise. | 322 | 3.75 | 1.06 | 0.55 | 0.72 |

*Note.* N differs by item due to participants selecting “not applicable” for specific items. Wording of items as administered in studies.
